# A Modified Reverse One-Hybrid Screen Identifies Transcriptional Activation Domains in PHYTOCHROME-INTERACTING FACTOR 3

**DOI:** 10.3389/fpls.2016.00881

**Published:** 2016-06-17

**Authors:** Jutta C. Dalton, Ulrike Bätz, Jason Liu, Gemma L. Curie, Peter H. Quail

**Affiliations:** Peter H. Quail Lab, Plant Gene and Expression Center, Department of Plant and Microbial Biology, University of California BerkeleyAlbany, CA, USA

**Keywords:** PHYTOCHROME INTERACTING FACTOR, transcriptional activation, yeast-one hybrid, loss of function screens, light signaling, phytochrome

## Abstract

Transcriptional activation domains (TADs) are difficult to predict and identify, since they are not conserved and have little consensus. Here, we describe a yeast-based screening method that is able to identify individual amino acid residues involved in transcriptional activation in a high throughput manner. A plant transcriptional activator, PIF3 (phytochrome interacting factor 3), was fused to the yeast GAL4-DNA-binding Domain (BD), driving expression of the URA3 (Orotidine 5′-phosphate decarboxylase) reporter, and used for negative selection on 5-fluroorotic acid (5FOA). Randomly mutagenized variants of PIF3 were then selected for a loss or reduction in transcriptional activation activity by survival on FOA. In the process, we developed a strategy to eliminate false positives from negative selection that can be used for both reverse-1- and 2-hybrid screens. With this method we were able to identify two distinct regions in PIF3 with transcriptional activation activity, both of which are functionally conserved in PIF1, PIF4, and PIF5. Both are collectively necessary for full PIF3 transcriptional activity, but neither is sufficient to induce transcription autonomously. We also found that the TAD appear to overlap physically with other PIF3 functions, such as phyB binding activity and consequent phosphorylation. Our protocol should provide a valuable tool for identifying, analyzing and characterizing novel TADs in eukaryotic transcription factors, and thus potentially contribute to the unraveling of the mechanism underlying transcriptional activation.

## Introduction

During the dark-to-light transition in the early stages of a plant's life, 10% of the genome experiences a change in gene expression, initiated by the phytochromes (phyA to phyE in Arabidopsis), the plant's red (R) and far red (FR) responsive photoreceptors (Tepperman et al., 2001). PIF proteins (Phytochrome Interacting Factors) are the main signal transduction route through which these gene regulatory changes are channeled (Leivar et al., 2009). The seven known PIF proteins (PIFs 1 and 3–8) are closely related basic helix-loop-helix (bHLH) transcription factors, which directly interact with phyB in a R-light-dependent manner (Ni et al., 1999; Khanna et al., 2004), the predominant phytochrome functioning in continuous light conditions. While phyB is cytoplasmically-localized in the dark (D), it migrates into the nucleus upon activation by R light (660 nm; Sakamoto and Nagatani, 1996; Kircher et al., 1999) which is essential for its function (Huq et al., 2003). However, PIF proteins are constitutively nuclear (Ni et al., 1998). They are able to hetero- and homodimerize, and bind DNA directly (Martínez-García et al., 2000; Toledo-Ortiz et al., 2003), by recognizing two variant hexameric motifs called a G-Box (CACGTG) and a PBE-box (CACATG; Zhang et al., 2013). PIFs have both, distinct and overlapping functions. Single *pif* mutants show rather subtle phenotypes in photomorphogenesis, but their functional redundancy becomes apparent in the *pif* quadruple mutant (*pif1pif3pif4pif5* called *pifq*), which shows a distinctive *cop*-like (Constitutive Photomorphogenic, COP) phenotype in D (Leivar et al., 2008). This observation demonstrates a central function of the PIFs in actively promoting skotomorphogenesis (Leivar et al., 2008). PIF1, 3, 4, and 5 all possess intrinsic transcriptional activation activity and are thus classified as transcriptional activators (Huq et al., 2004; Al-Sady et al., 2008; de Lucas et al., 2008). In light, transcriptional activation is suppressed by phyB, which induces rapid phosphorylation of multiple sites in PIF3 (Ni et al., 2013). This phosphorylation promotes recruitment of LRB-E3 ubiquitin ligases, which polyubiquitinate both PIF3 and phyB proteins, triggering their subsequent degradation by the 26S proteasome (Ni et al., 2014). This mutually assured degradation mechanism both regulates PIF-target-gene expression and attenuates light signaling by limiting the plant's light perception capacity during photomorphogenesis.

The major pathway for transcriptional activation of protein-encoding genes in eukaryotes, is through the recruitment of RNA polymerase II and its initiation complexes to promoter elements in the genome. In the simplest model of transcriptional activation, transcription factors such as PIF3 occupy specific promoter sequences by directly or indirectly binding DNA, and simultaneously binding transcriptional co-activators, thereby directing the basal transcriptional machinery to the target genes (Sainsbury et al., 2015). The interaction between transcription factors and components of the pre-initiation complex (PIC) is facilitated by TADs. Binding of a transcription factor to a co-activator initiates a cascade of events, including possible chromatin restructuring, in addition to recruitment and assembly of the RNA Pol II PIC, and successful transcript elongation (Weake and Workman, 2010). Chromatin remodeling, through post-translational modifications of histones, such as methylation, phosphorylation, acetylation, and ubiquitination are known to activate or repress transcription. For instance, di-methylation at position K4 in Histone 3 is a known permissive mark (Zhang et al., 2009), whereas di-methylation at position K9 in the same Histone is repressive (Zhou et al., 2010). Transcription factors are an important mediator for this transcriptional activation mechanism, since they can recruit or direct chromatin modifying enzymes to specific sites in the genome. The mechanism of PIF activation of target-gene transcription (most prominently in dark-grown seedlings where PIFs are most abundant) has not been defined. However, there is evidence that PIF3 can repress expression by interacting with a histone deacetylase (HDA15). This interaction results in repression of several genes involved in chlorophyll biosynthesis and photosynthesis, in dark-grown seedlings, and is reversed upon light-induced degradation of the PIF3 protein (Liu et al., 2013).

TADs provide the contact surface for the recruitment of either chromatin remodeling proteins or the basal transcription machinery. Functional TADs remain difficult to predict or define, as their sequences are not obviously related and share no unifying structural element (Hope and Struhl, 1986; Sigler, 1988; Brzovic et al., 2011). Additionally, residues in identified TADs can often be replaced with little to no loss of activity. For example, exchanging leucine 439 and 444 with any other bulky hydrophobic side chain shows minimal loss of activity in the herpes virus activator VP16 (Regier et al., 1993), although the functional importance of these residues becomes apparent when exchanging them with alanine, which does not have similar chemical properties. Only residue F442 is critical in VP16 and cannot be replaced without loss of activity by any other amino acid (Cress and Triezenberg, 1991; Regier et al., 1993), indicating a minimal sequence specificity for transcriptional activation (Warfield et al., 2014).

Historically, TADs have been classified according to the chemical properties of their predominant amino acid (aa) composition, such as acidic, glutamine-rich, or proline rich (Mitchell and Tjian, 1989; Johnson et al., 1993), although this classification does not reflect the functional importance of these aa. In acidic domains, as long as a sufficient level of acidity is retained, individual residues appear not to be crucial (Cress and Triezenberg, 1991). Rather the position of hydrophobic residues within these domains are important for function (Cress and Triezenberg, 1991; Blair et al., 1994; Drysdale et al., 1995; Sainz et al., 1997). The NMR structure of one such acidic transcriptional activator from yeast, Gcn4, with its binding target, mediator subunit Gal11, shows only four hydrophobic aa to be responsible for the contact between the two proteins, supplying a very simple interaction surface (Brzovic et al., 2011; Warfield et al., 2014). Sequences as short as 9–10 aa have been found to be autonomous activators, enabling detailed analysis of their structures (Blair et al., 1994; Piskacek et al., 2007; Warfield et al., 2014) and defining a prediction algorithm (Piskacek et al., 2007). However, many transcriptional activators are not functional if isolated without protein context and identification and characterization of TADs still rely on functional analysis *in vivo*. Since the transcriptional machinery and co-activators are conserved in eukaryotes (Yanagisawa, 2001; Srivastava et al., 2015), many TADs are functional across species, even though their target genes are not conserved (Sadowski et al., 1988). This enables the use of yeast screening methods for TAD analysis (Sainz et al., 1997; Yanagisawa, 2001; Tiwari et al., 2012).

In a common approach, fusing a known TAD to the GAL4-DNA binding domain has enabled the detailed analysis of TADs (Cress and Triezenberg, 1991; Sainz et al., 1997; Yanagisawa, 2001). Typically amino acid substitutions in such fusion constructs have been analyzed for loss of function by quantitative LacZ assays (Wu et al., 1996; Sainz et al., 1997; Serpe et al., 1999). However, this assay method is laborious and limits throughput. Here, by combining a random mutagenesis strategy with a negative selection screen provided by URA3 + 5FOA, instead of the LacZ colorimetric assays, we were able to identify and characterize residues in PIF3 responsible for transcriptional activation, in a high throughput manner. A similar reverse 1 hybrid configuration has been successfully used to eliminate transcriptional activators from a library of fusion proteins (Walhout and Vidal, 1999), but the specific configuration we describe has, to our knowledge, not been previously explored for identification of TADs and loss-of-function analysis. Furthermore, we present a strategy to minimize false positive detection that is adaptable to reverse yeast-2-hybrid screens as well. With this method, we have successfully identified novel, unpredicted and non-autonomously active TADs in PIF3.

## Materials and methods

### Cloning of reverse yeast-1-hybrid vector pBLAU

The vector pBRIDGE was first converted into a C-terminal fusion vector pBC. This was done, by amplifying a fragment from pBRIDGE with primer MZ380/MZ381 (see Supplemental Table 1), which introduces a PacI and SacI site before the BD, while keeping the HindIII site, as well as inserting a stop codon behind the BD with a SpeI site. A second fragment was amplified from pBRIDGE with primers MZ382 and MZ383 with SpeI and HindIII sites to reconstitute the selection marker, which is partially excised during the cloning process. Both fragments were subcloned into pCR2.1-TOPO/TA (Invitrogen), subsequently digested with SpeI, leading to excision of MZ380/381 fragment and opening of subclone MZ382/383. The purified MZ380/381 fragment was ligated into subclone MZ382/383 and selected for orientation to create subclone pBDTrp. The recombined fragment BDTrp was excised with HindIII and ligated into HindIII-opened pBRIDGE, creating pBC. To create pBAC, the full-length ADH1 promoter from pGBK (Clontech) was amplified with primers JR137/JR138 and inserted via SacI digestion and ligation into pBC. Vector pBAC0 was then created from pBAC, by removing the BD via PacI/SpeI digest and replacing it with a PacI/SpeI PCR fragment created from pBAC with primers JR189/JR190. Inserting AUR1C fragment from pAUR123 (Clontech), amplified with primers JR191 and JR192 via SpeI/SalI into pBAC0, gave rise to pBACALAU. pBACALAU features the Gal4BD and AUR1C as an in frame fusion, with a 10 aa linker in between. Finally, pBLAU was cloned, by exchanging the pBACALAU PacI/SpeI fragment with a PacI/SpeI fragment amplified via PCR from pBACALAU with JR195 and JR196. PIF3, PIF1, PIF4, and PIF5 were inserted into SmaI-opened pBLAU from fragments with the following primers: PIF3 JR200/JR201, PIF1 JR226/JR227, PIF4 JR228/JR229, and PIF5 JR230/231.

### Cloning of pGAD and pBAC-N

pBAC-N was created by inserting the N-terminal 621 aa of phyB via PacI digestion into the vector pBAC (see above). Amplification of the phyB-N fragment for this purpose was achieved with primers JR171 and JR172. All PIF variants were cloned into pGAD via Gateway technology according to the manufacturer's recommendations. ENTRY vectors were created using the pCR8/GW/TOPO/TA cloning Kit (Invitrogen) and a Gateway compatible pGAD vector was kindly provided by Jaume Martinez-Garcia.

### Yeast transformation

All yeast transformations were done, using the PEG/LiAC method (Gietz and Schiestl, 2007).

### Error prone PCR and homologous recombination

Error prone PCR was performed with GeneMorph II Random Mutagenesis Kit (Agilent) according to the manufacturer's instructions for a low mutation rate. JR197 and JR198 were used as primers and the final PCR reaction was DpnI digested and subsequently precipitated with 3 M NaAc and Ethanol. pBLAU was SmaI digested, dephosphorylated, and also precipitated via NaAc/Ethanol. For homologous recombination, yeast strain MaV103 was transformed using a 1:1 ratio of pBLAU and PIF3-PCR product.

### Reverse yeast-1-hybrid screen and selection

Reverse yeast-1-hybrid screening was performed with strain MaV103 (Vidal et al., 1996) on selection plates containing –W synthetic dropout medium (SD), 0.035% 5FOA, 0.00001% Aureobasidin A (AbA). In a first selection, positive colonies were restreaked on three different plates either containing –W SD, or –W/U SD, or –W/5FOA/AbA. Only those colonies showing no growth on –W/U, but good growth on both other plates were subjected to plasmid extraction. Plasmids from individual colonies were extracted via Lyticase digest and glass beads with a subsequent Phenol extraction. All plasmids were then electroporated into *E. coli* (XL1 blue) cells and purified via Miniprep (Quiagen), before subjecting them to a second selection process. All remaining plasmids were individually back transformed into MaV103 and selected again on the three different media –W, –W/U/AbA, and –W/5FOA/AbA. Those plasmids which showed good growth on –W and –W/5FOA/AbA after back transformation were finally subjected to sequencing.

### Site directed mutagenesis

Site directed mutagenesis was performed with the QuickChange II XL site-directed-mutagenesis-kit (Agilent) according to the manufacturer's instructions. Primers used for site directed mutagenesis are listed in Supplemental Table 1.

### Quantitative β-gal assays

For quantitative β-Gal assays, plasmids were transformed into yeast strain Y187 (Clontech). Assays were performed as described (Clontech Yeast Protocols Handbook). Liquid quantitative interaction assays between PIF variants and phyB-N were performed as described (Shimizu-Sato et al., 2002). Each analysis was performed with at least three biological replicas and at least two technical replicas. As a negative control pBacalau was used, which was subtracted from all the samples, before normalization. As we observed high variability in PIF3 expression and activity and were unable to obtain full length PIF1 or PIF3 on a Western blot, we cloned PIF1, PIF3, and both their variants into the pEG202 vector and did the quantitative analysis in yeast strain EGY48 with reporter pSH18−34. As a negative control, a LexA-GFP fusion was used.

### Yeast-2-hybrid plate growth assays

Light dependent interactions between PIF variants and the phyB N-terminus were tested in a yeast-2-hybrid assay using the strain AH109 (Clontech) pre-transformed with pBAC-N. Cell concentration was determined by measuring OD_600_ spectrophotometrically (BioRad SmartSpec, 3000). Selection plates contained –W/L/H, 1 mM 3AT, and 40 μg/ml X-α-Gal. R light was provided by filtered fluorescent tubes, described in Parks and Quail (1993) at a fluence rate of 0.9 μmoles m^−2^ s^−1^.

### Protein extraction

1.5 ml yeast liquid cultures were sedimented by centrifugation, washed one time with sterile water, and incubated in 1.5 ml 0.1 M NaOH for 10 min at RT. Cells were collected, supernatant removed, and resuspended in 100 μl modified 2x Laemmli buffer containing 10% β-MeEtOH. Following a 3 min boil in a waterbath, cells were again pelleted and protein concentration determined with Amidoblack.

### Protein concentration measurements

One to ten microliters of protein sample in Laemmli buffer were diluted with water to 200 μl total volume and precipitated through the addition of 800 μl Amidoblack stain (90% Methanol, 10% acetic acid, 0.005% Naphtol blue black). Samples were centrifuged at 12,000 g for 10 min at 4°C and the supernatant discarded. Samples were washed once with Destain solution (90% Methanol, 10% acetic acid) and the supernatant thoroughly removed. Pellets were air dried quickly and resuspended in 250 μl 0.2 M NaOH. A_620nm_ of 200 μl from each sample and standard was determined in a microplate reader (SpectraMax190, Molecular Devices) and finally plotted against a standard curve. All samples were analyzed in duplicates.

### Western blots

Twenty-five to Hundred micrograms of total protein was loaded into each lane and separated on a 8% Polyacrylamide Gel, before transferred to PVDF membrane. Gal4 BD was probed with αBD antibody (RK5C1, Santa Cruz Biotechnology) at a 1:5000 dilution and a secondary αmouse IgG antibody (Promega) coupled to HRP. LexA was probed with monoclonal LexA antibody (sc-7544, Santa Cruz) also in a 1:5000 dilution and the same secondary antibody. Detection was finally performed with ECLprime (Amersham) substrate and standard X-ray film. The Western blot signal was quantified with the Image Quant Software (Life sciences) by analyzing two different exposures for each blot in the linear range.

### Alignments

Alignments between PIF1, PIF3, PIF4, and PIF5 was done using the entire protein sequence of the PIFs and aligning them with the ClustalW2 algorithm using a Gonnet matrix. Putative PIF3 homologs in other plant species were identified using either full protein sequence or aa 90–120 in a BLAST search. Sequences were downloaded and subsequently aligned, using only the relevant 30 amino acids as input for ClustalW2.

## Results

### Improving false positive detection rates of reverse yeast-1-hybrid methods with a second selectable marker and an out of frame insertion site

**Figure 1:**
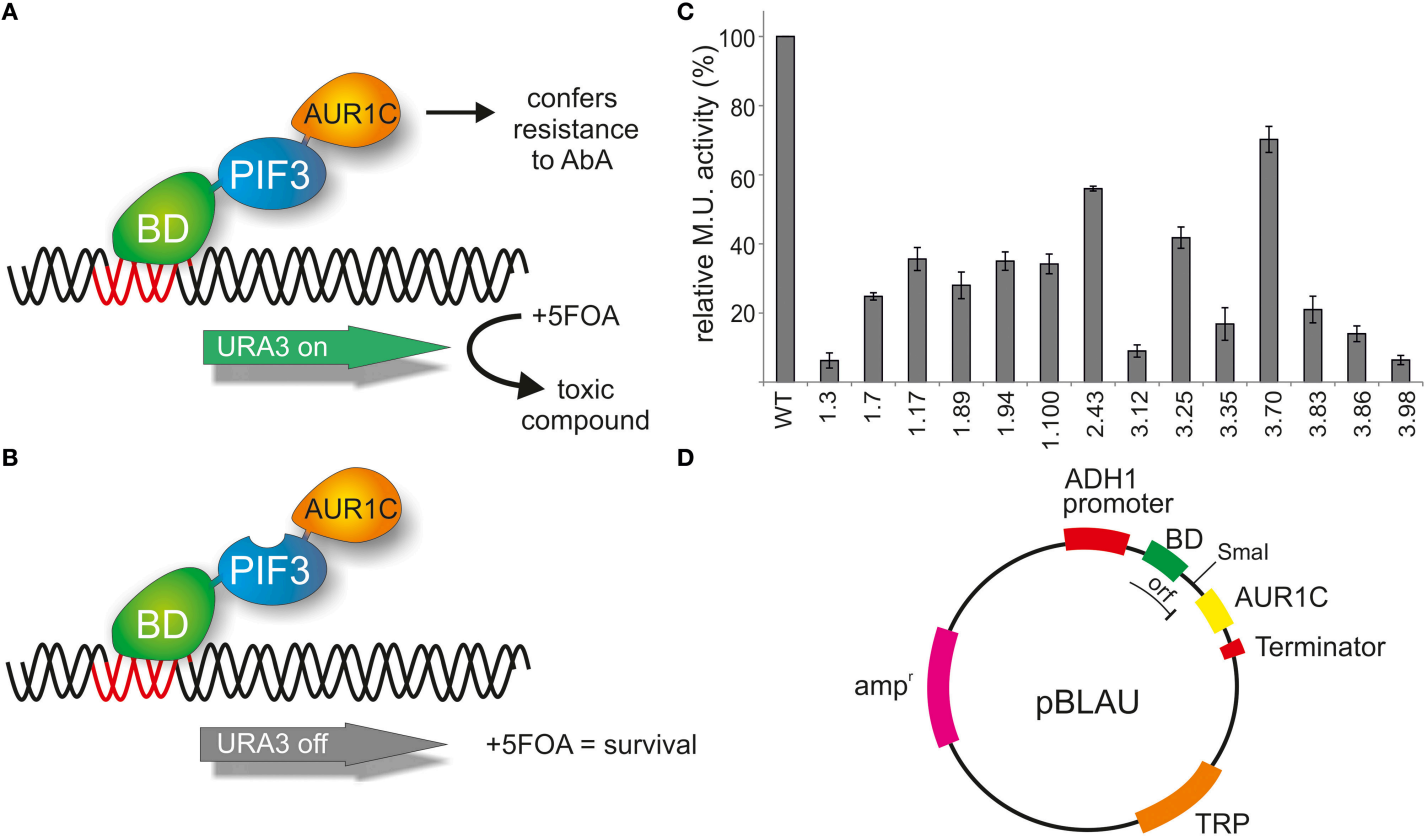
**Reverse yeast-1-hybrid scheme for TAD identification. (A)** PIF3 is fused N-terminally to the DNA binding domain (BD) of the yeast GAL4 transcription factor and C-terminally to AUR1C, which confers resistance to AbA. AUR1C serves as a second selectable marker, providing verification of the expression of the fusion construct, and thus reducing false positives, such as premature stop codons, frameshift mutations, or low-expression clones. The PIF3 transcription factor self-activates transcription of the reporter gene URA3 in yeast. URA3 converts 5FOA (Fluroorotic acid) into a toxic compound, leading to negative selection. **(B)** In the screen, randomly mutagenized PIF3 sequences were inserted between BD and AUR1C. If the mutagenized PIF3 fails to activate transcription, URA3 will not be expressed, leading to survival in the presence of 5FOA. **(C)** Relative transcriptional activity (rel. Miller units) of 14 randomly selected colonies identified with the rY1H method. **(D)** Schematic representation of the rY1H vector pBLAU. The coding sequences of BD and AUR1C markers are out of frame, to reduce background of intact pBLAU plasmid under screening conditions. Insertion of the protein of interest into the SmaI opened vector pBLAU is done via homologous recombination. The reading frame of the fusion protein is restored by adding an additional base in the reverse primer used for homologous recombination.

The reverse 1-hybrid configuration presented here uses the intrinsic activation activity of a protein to map residues responsible for transcriptional activation. By using the negative selection provided by URA3 expression in the presence of 5FOA, randomly mutagenized PIF3 variants with lack, or low levels, of transcriptional activity can be selected for. In order to exclude false positives, such as premature stop codons, frameshift mutations, and low expressing colonies, an additional selection marker AUR1-C (Aureobasidin resistance) was inserted into the method (Figure 1A). The presence of AbA leads to growth arrest of yeast cells, but it can be overcome by expressing AUR1-C. Therefore, a fusion of AUR1-C at the C-terminal end of PIF3 serves as a control for expression of the full-length fusion protein (BD-PIF3-AUR1C) in the presence of AbA (Figure 1B).

The PIF3 sequence was randomly mutagenized using error prone PCR and subsequently inserted into vector pBLAU via homologous recombination. As BD and AUR1C are out of frame in pBLAU, the empty vector is not selected for (Figure 1D). In order to reconstitute the reading frame, an additional base was added to the PIF3 insert. To confirm the properties of pBLAU and its derivatives, transformed yeast was plated on positive and negative selection media (Table 1) and compared to yeast transformed with pBACALAU, an in-frame fusion of BD-AUR1C.

**Table 1:** **Growth phenotypes of the vectors used for rY1H screening**.

**Vector**	**Fusion protein**	**–W (%)**	**–W/U (%)**	**–W/U/AbA (%)**	**–W/5FOA (%)**	**–W/5FOA/AbA (%)**
pBLAU	BD	100	0	0	69	0
pBACALAU	BD-AUR1C	100	0	0	96	20
pBLAU-PIF3	BD-PIF3-AUR1C	100	56	60	8	0

Yeast transformed with pBLAU and pBACALAU were both unable to grow on positive selection medium (−W/−U) regardless of the presence of AbA (Table 1). Conversely, yeast transformed with pBACALAU is able to grow on both negative selection media, with or without AbA (Table 1). However, yeast transformed with pBLAU is not able to grow on negative selection media in the presence of AbA, confirming that the empty vector is not selected under screening conditions (Table 1). Although strongly reduced, there still is measurable growth of pBLAU-PIF3 on negative selection medium without AbA that cannot be suppressed by increasing concentrations of 5FOA. This background is eliminated if AbA is additionally added to the medium.

### Screening of PIF3 for amino acid residues involved in transcriptional activation

Molecularly, PIF3 acts as a transcriptional activator in plants, actively promoting skotomorphogenesis in D (Leivar et al., 2008). When fused to the GAL4-BD, PIF3 is likewise able to induce expression of the URA3 reporter gene in yeast (Table 1), leading to survival on selection plates lacking Uracil. Alternatively, on negative selection medium containing 5FOA, no colonies expressing BD-PIF3 could be detected, thus confirming an intrinsic transcriptional activation activity for PIF3 in yeast (Table 1).

Taking advantage of this negative selection phenotype on 5FOA plates, PIF3 was randomly mutagenized and the resulting variants screened *en masse* for survival, which indicates a loss of transcriptional activation activity (Figure 1B). A total of 1.5 × 10^6^ colonies were screened and 304 colonies were selected on negative selection plates. To further reduce the possibility of false positives, all 304 colonies were subjected to two further reselection steps, removing ambiguous colonies, which could grow on both positive and negative selection medium, as well as those colonies with only weak or slow growth on negative selection medium. From this, 65 colonies remained. An incomplete and random analysis of colonies that were removed through both selection steps showed a prevalence for multiple plasmids in these cells, likely leading to the observed phenotypes. Out of the 65 remaining colonies, 14 were randomly selected and their transcriptional activity quantified (Figure 1C). All colonies showed strongly reduced transcriptional activity, varying between 5 and 65% of the WT activity. This suggests that our method was successful in selecting PIF3 variants with significantly reduced transcriptional activation activity (Figure 1C).

All 65 remaining colonies were subsequently sequenced and analyzed for mutations. Noteworthy, the full-length insertion of PIF3 could be identified in all plasmids. However, due to sequencing quality issues, only 53 sequences were used for further analysis. These sequences contained a total of 235 base pair changes, 173 of which result in an amino acid substitution (non-synonomous mutations) and 62 were silent (synonomous) mutations. Each clone, on average, thus contained three non-synonomous and one silent mutations, with a maximum of 12 mutations detected in a single clone. Also, every clone had at least one non-synonymous mutation, suggesting that our selection strategy was indeed powerful enough to remove false positives from the analysis.

### PIF3 has two TADs, both necessary but not sufficient for transcriptional activation

**Figure 2:**
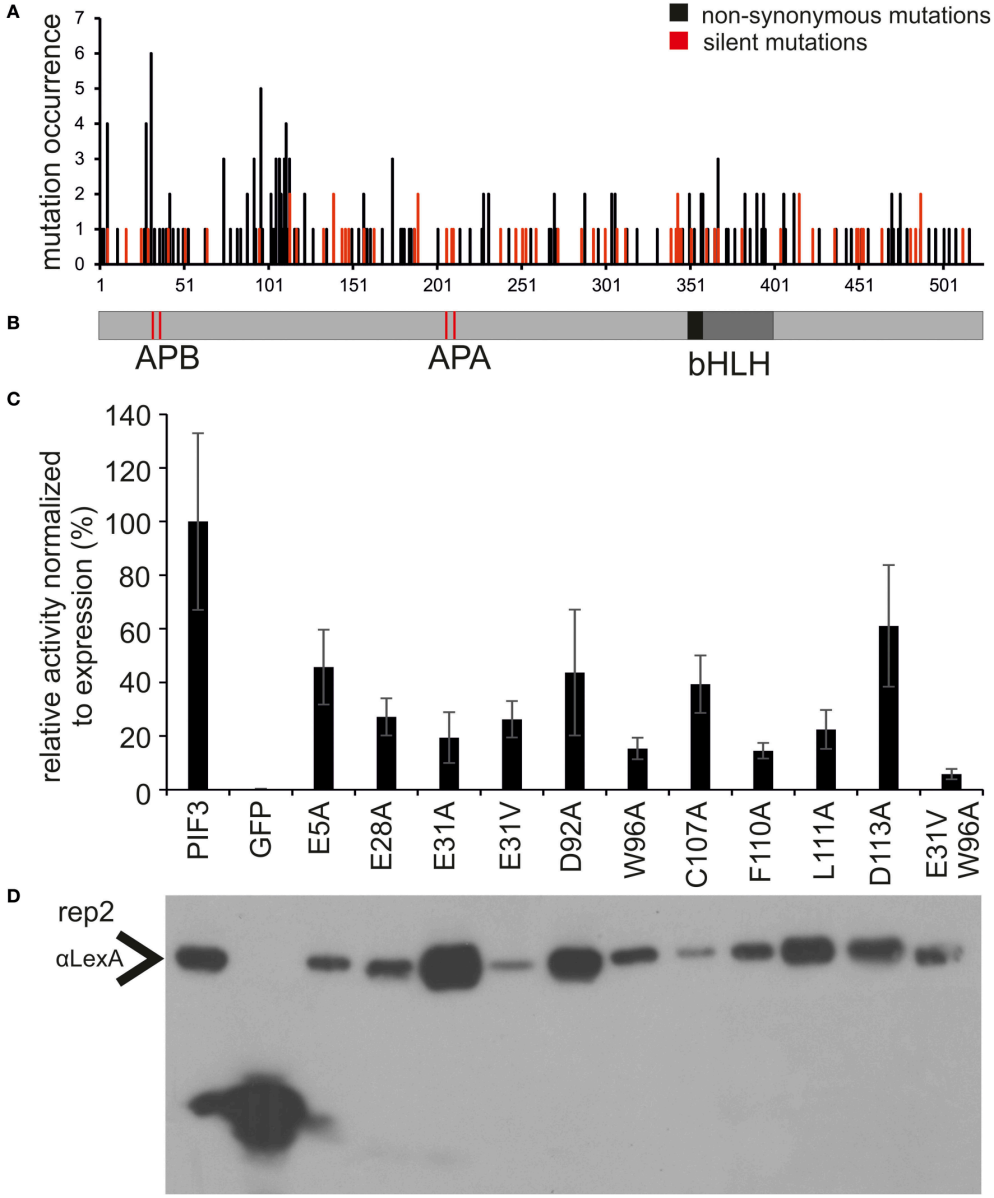
**Identification and characterization of transcriptionally impaired PIF3 variants. (A)** Distribution of mutations affecting transcriptional activity across the PIF3 protein shown underneath in panel **(B)**. Shown in black are the mutations leading to a non-synonomous amino acid substitution, while silent mutations are marked in red. The frequency of non-synonomous mutations is strongly increased in two discrete regions in the PIF3 N-terminal region. **(B)** Schematic representation of the PIF3 protein sequence and its known features to scale with the sequence length in panel **(A)**. **(C)** Relative Miller unit (M.U.) activity of single amino acid substitutions in PIF3 as determined by quantitative b-Gal assay and normalized to protein expression by quantitative Western blot analysis. Measurements represent the mean value of three biological and two technical replicas each. Standard error of the mean is indicated by error bars. All residues selected in this analysis were identified in the screen by high mutation occurrence. Amino acids are indicated in standard single letter code: first letter gives the original aa identity, number gives the position in the protein sequence, last letter gives the mutant aa residue. **(D)** The Western blot corresponds to replica 2 in Supplementary Figure 1, which depicts the raw data for normalization of the activity levels.

Since every sequence contains on average more than one mutation, amino acids affecting transcriptional activation might be obscured by the simultaneous occurrence of unrelated mutations. Therefore, the occurrence of all non-synonymous and silent mutations was mapped onto the PIF3 primary sequence (Figures 2A,B). Since, there has been no selection pressure on the silent mutations, we expected them to be evenly distributed over the entire molecule. Indeed, the distribution of silent mutations occurred very evenly over the entire molecule and mutation frequency is very constant. By contrast, the non-synonymous mutations cluster in two discrete regions at the N-terminus, from aa 27–42 to 90–120, characterized by a dramatically increased mutation frequency, comprising both an increased number of mutated residues per total number of residues in this region, and a high occurrence of multiple mutations in individual residues (high amplitude; Figure 2A). One of these putative TADs physically overlaps with the APB (Active phyB Binding) domain, required for phyB binding (Figure 2A). Furthermore, a third region with a high amplitude was identified, but since it appeared to be an isolated single residue without the accompanying elevated regional mutation frequency, residue E5 was not classified as a motif or domain. Nevertheless, it certainly might be part of a bipartite or even tripartite TAD that requires folding to bring the separate residue into a domain context.

**Figure 3:**
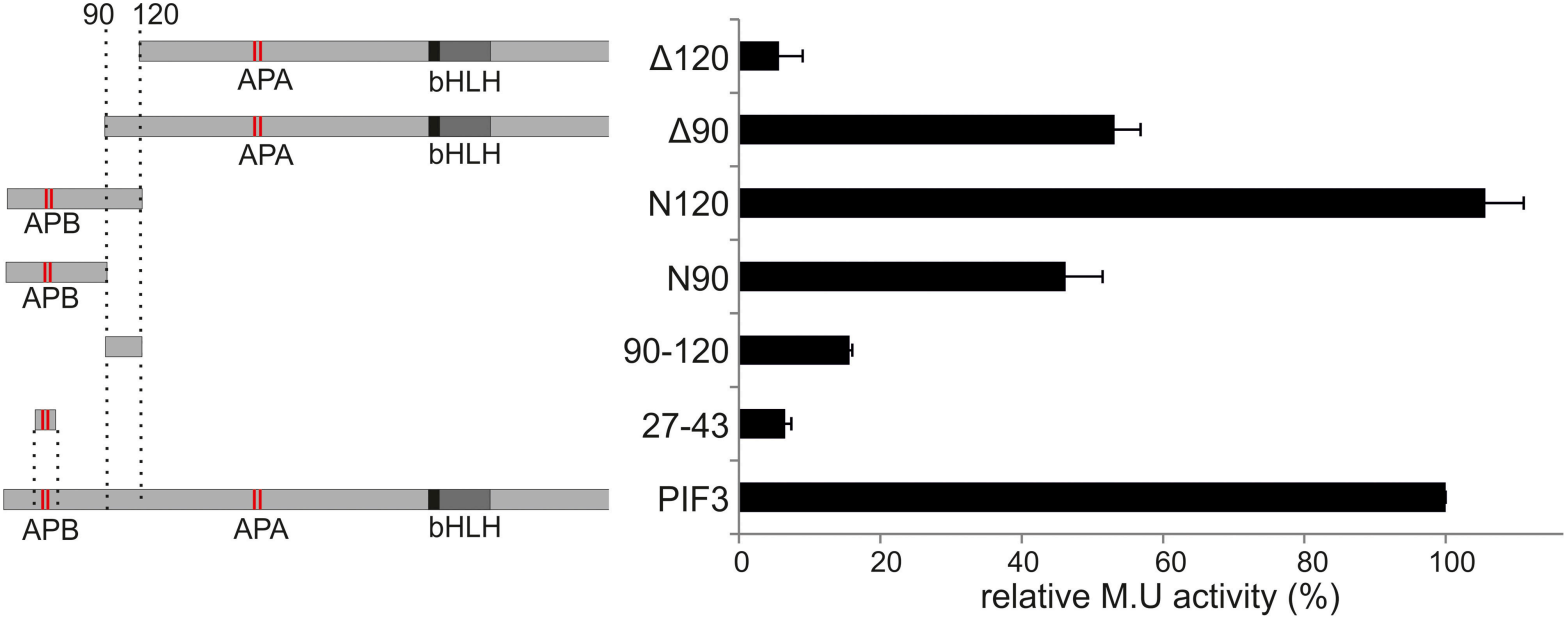
**Deletion construct analysis**. Relative transcriptional activity of PIF3 and six deletion constructs. **Left:** Schematic representation of the constructs. **Right:** b-Gal activities, relative to full-length PIF3. Error bars represent standard error of the mean.

To elucidate the role and influence of the individual putative TADs, a deletion analysis was performed (Figure 3). Neither one TAD by itself showed significant transcriptional activation capacity when expressed alone (Figure 3), indicating that each one individually is not sufficient to activate transcription. On the other hand, the complete 120-aa N-terminal peptide sequence, containing both domains, was sufficient to reconstitute full WT transcriptional activation activity (Figure 3), indicating that a larger protein context, or possibly a specific fold/tertiary structure might be important for the promotion of transcription. Only half the transcriptional activity was restored by expressing the N-terminal 90 aa, and comparable activity was seen, if the first 90 aa were deleted (Figure 3). By contrast, deletion of the first 120 aa, almost completely abolished transcriptional activation capacity to 5% of the WT (Figure 3). This result indicates that both TADs are necessary and contribute approximately equally to the transcriptional capacity of the WT PIF3 and are therefore acting as independent modules.

### Amino acid substitutions specifically affect TA function

**Figure 4:**
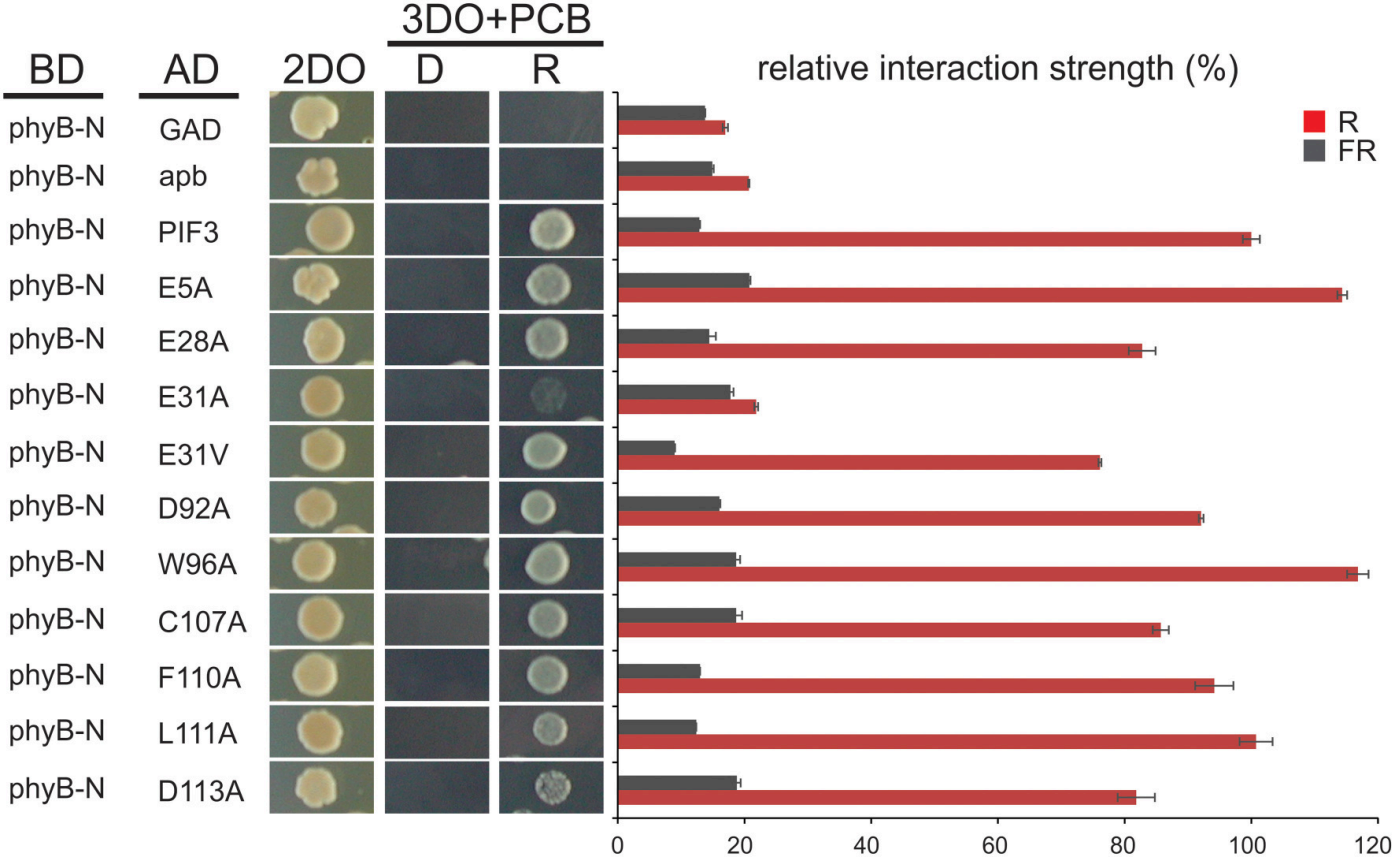
**Interaction of PIF3 variants with phyB**. Light dependent yeast-2-hybrid growth assay on selection plates and quantitative b-Gal assay to determine the interaction strength of the PIF3 variants with photoactivated phyB. Despite reduced transcriptional activity of all PIF3 variants shown, interaction with phyB is largely unaffected and remains strictly R-light dependent. Error bars represent standard error of the mean. 2DO indicates −W/L dropout medium, 3DO indicates −W/L/H dropout media with 1 mM 3AT.

To investigate the effect of individual amino acids on transcriptional activation, the residues with the highest mutation occurrence were individually exchanged with an alanine by site-directed mutagenesis (Figure 2C). Residue E31 was additionally mutated into a valine, an exchange that was found in the screen multiple times in this position. Because we observed variation in the level of PIF-protein expression among the different mutant sequences (Figure 2D), we normalized the yeast-assay β-Gal activity levels to the corresponding PIF-protein level (determined by quantitative immunoblot analysis), and plotted those values relative to the wild-type PIF3 sequence value (Figure 2C and Supplementary Figure 1). As a reduction in transcriptional activity could be due to improper protein folding that would generally affect all PIF3 functions, light dependent interaction with phyB was also tested in all single residue PIF3 variants (Figure 4). Transcriptional activity was indeed reduced in all variants to about 5–60% of the wildtype, confirming their involvement in this function. Interaction with phyB remained unaffected by the mutations E5A, D92A, W96A, F110A, and L11A, indicating a very specific defect of these residues in promoting transcription, rather than an overall folding issue. Variants E28A, C107A, and D113A still interact with phyB in a light-dependent fashion, but they display a mildly reduced interaction strength (Figure 4), and a moderate reduction of transcriptional promotion to a level that is between 40 and 60% of the WT activity (Figure 2C). Out of the tested variants, E31V, W96A, and F110A show the most dramatic influence in lowering transcriptional activation (Figure 2C). The double mutation, E31V/W96A, almost completely abolished transcriptional promotion, with < 10% of the WT transcription levels remaining (Figure 2C). The effect of W96A and F110A on transcription is very selective, since both variants display normal or slightly enhanced interaction with phyB (Figure 4). Mutations in the APB motif, E31A, and E31V, show distinct phenotypes in both responses. E31A does reduce transcriptional reduction as strongly as E31V. At the same time, E31A also does not interact with phyB, showing no growth on selective media in a yeast-2-hybrid assay, whereas E31V is well-able to interact with phyB though the interaction strength is reduced to 75%. Thus, the phenotypes of E31A and E31V confirm a dual functionality of residue 31, in both transcriptional promotion and phyB binding.

The PIF3 molecule contains 72 serines and 23 threonines, making serines by far the most prevalent amino acid in its primary sequence. At least 26 of these were previously identified as being phosphorylated in either D or R (Ni et al., 2013), and 4 of these 26 were identified as being mutated more than once in our screen: S88, S102, S108, and S174. Interestingly, S88, S102, and S174 are phosphorylated in a strictly R light dependent manner, whereas S108 is already highly phosphorylated in D, indicating a partial overlap between phosphorylation and transcriptional activation potential.

### Functional conservation of TADs in other PIFs and across species

**Figure 5:**
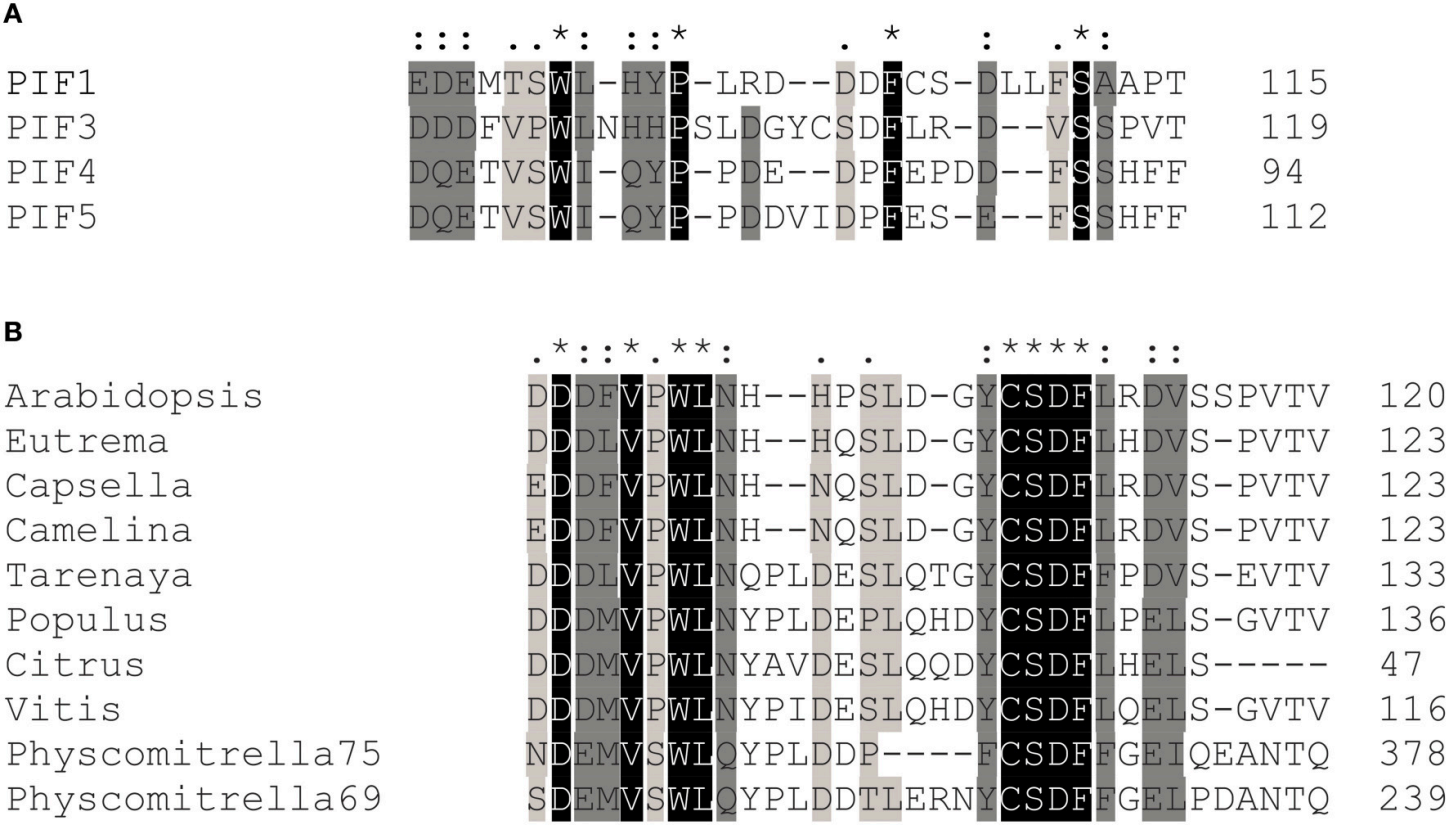
**Transcriptional activation domain sequences are conserved among the PIFs. (A)** Sequence alignments show that key residues in the transcriptional activation domain of PIF3 are conserved in PIF1, PIF4, and PIF5, as well as across species **(B)**. Asterisks indicate a fully conserved single amino acid; colons indicate conservative substitutions; periods indicate non-conservative substitutions.

After successful identification and characterization of individual amino acids, we were interested whether our screen had the power to predict similar TAD in other transcriptional activators. The APB motif has been shown to be conserved among PIF1, PIF3, PIF4, and PIF5 and alignments show that the key residues in the 90–120 TAD are highly conserved as well (Figure 5). Furthermore, by using the 90–120 aa of PIF3 as an input for BLAST, we could identify several PIF3-like proteins in other species (Figure 5), which equally show a conservation of this domain, reaching as far back as Physcomitrella (Figure 5). Of particular interest are the residues W96 and F110, since these displayed the strongest influence on transcriptional activation (Figure 2C). Both residues are fully conserved in PIF1, PIF4, PIF5, and PIF3 as well as across species, supporting their importance for transcriptional promotion.

**Figure 6:**
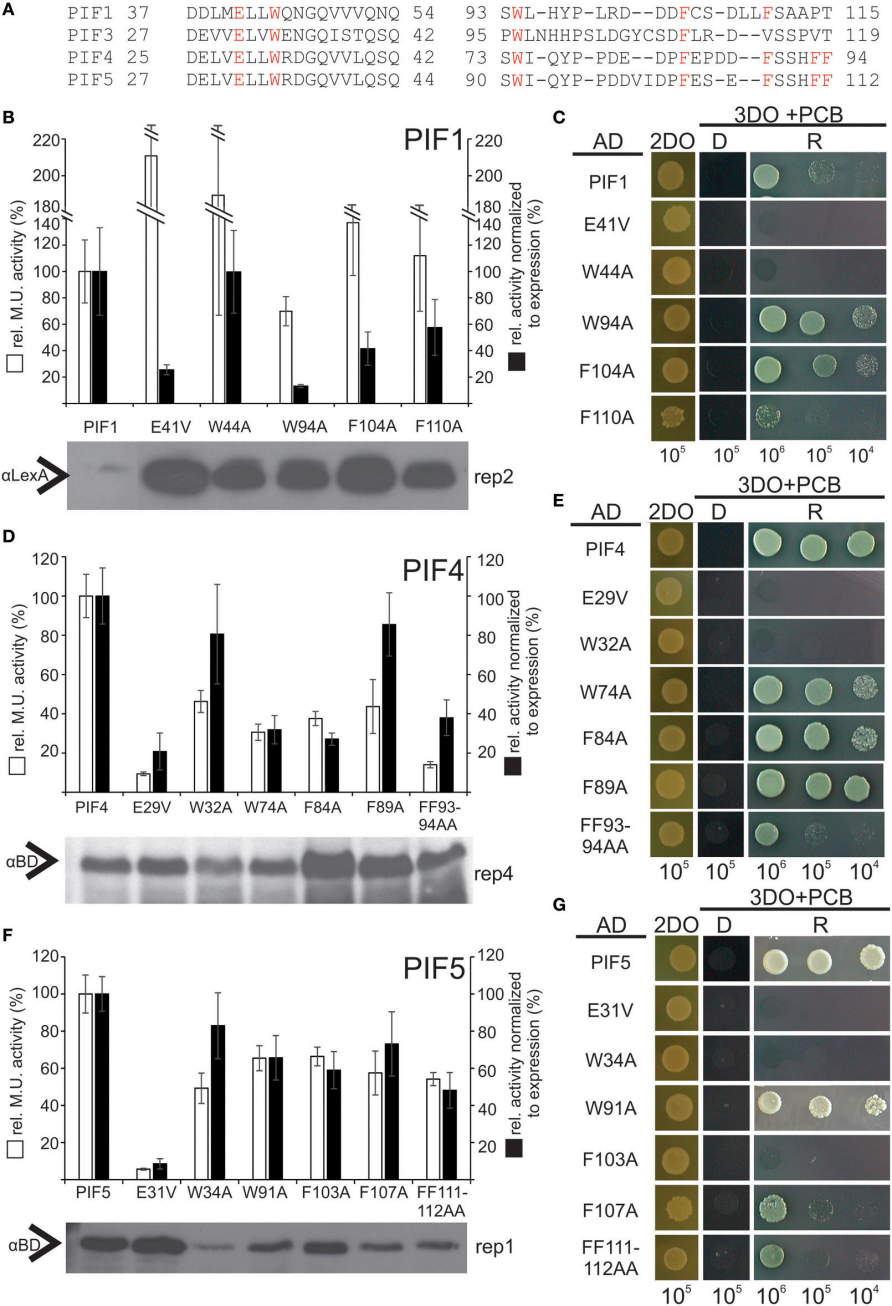
**Relative transcriptional activity and phyB-interaction capacity of PIF4 and PIF5 variants. (A)** Alignment of the two identified TADs in PIF1, PIF3, PIF4, and PIF5. Homologous residues used for mutagenesis are marked in red. **(B,D,F)** Site-directed mutagenesis of conserved residues in PIF1, PIF4, and PIF5 as measured by quantitative β-Gal assays. White bars represent the relative Miller unit activity in % and black bars represent the measured activity normalized to the expression levels, measured by Western blot relative to the wt PIF. Data is the mean of at least three biological replicas and three technical replicas each, normalized to two different exposures of Western blots, error bars indicate standard error of the mean. Representative Western blot underneath shows the expression of the fusion proteins and the corresponding replica number is given for each blot. The data, from which this figure is derived, is shown in Supplementary Figures 2–4. **(C,E,G)** Yeast-2-hybrid growth assays on selective media using the phyB-N terminus as a bait. D = dark-incubated; R = red light-incubated. 2DO indicates −W/L dropout medium, 3DO indicates −W/L/H dropout media with 1 mM 3AT. The total numbers of cells spotted are given below the panel.

To test the hypothesis that transcriptional activation is also functionally conserved among PIFs at key residues, we exchanged homologous residues in PIF1, PIF4, and PIF5 via site directed mutagenesis. Since hydrophobic residues showed the strongest phenotype for PIF3, we also included non-homologous W and F residues in the regions of interest (Figure 6A). All variants indeed displayed reduced transcriptional activation to varying extents, (Figures 6B,D,F; Supplementary Figures 2–4). This confirms a functional conservation among PIFs of these key residues. Since reduction of transcription could be due to diminished expression in the yeast, Western blots were performed to control for this possibility, and the quantified band intensities were used to normalize the β-Gal assay values to the protein expression levels (Figures 6B,D,F). Mutations in the APB domain, produced the strongest reduction in transcriptional activation for PIF1, PIF4, and PIF5, but also abolished light dependent interaction with phyB completely (Figures 6C,E,G). Unlike PIF3, exchange to a valine instead of an alanine in the APB domain does not reconstitute direct phyB interaction in PIF1, PIF4, or PIF5 (Figures 6C,E,G). In PIF4, W74A, and F84A (the equivalent to W96 and F110 in PIF3) show a considerable reduction to 40% wt transcriptional activity, whereas the same residues in PIF5, still show 70% transcriptional activity (Figures 6B,F). Generally, all residues within the second TAD in PIF5 do not appear to have a major influence on transcriptional activation (Figure 6F), which could indicate a possible shift in dominance for transcriptional activation toward the APB motif. Additionally, PIF5 is seemingly very sensitive to exchanges in general, as interaction with phyB also appears to be reduced in all variants except W91A (Figure 6G). PIF1 shows a very similar picture to PIF3, in which E41V, and W94A in PIF1 (equivalent to E31V and W96A in PIF3) displayed the strongest reduction in transcriptional activity (Figure 6B and Supplementary Figure 2). Noteworthy, we were unable to detect full length PIF1 or PIF3 on a Western blot using αGAL4-BD, only a shorter degradation product of distinct length could be detected. This negative selection for full length PIF1 and PIF3 in yeast cells, which is also accompanied by a severe growth retardation and observably smaller cells could be due to squelching, an inhibitory effect observed for strong activators (Gill and Ptashne, 1988).

## Discussion

### Expression marker AUR1C eliminates false positives

Reverse yeast-2- and 1-hybrid screens can be directed toward selecting loss-of-function mutations in a randomly mutagenized protein, using negative selection provided by URA3 expression in the presence of 5FOA (Vidal et al., 1996). The classical reverse yeast-1-hybrid screen configuration identifies DNA motifs that are bound by a transcription factor of choice. However, it can also be employed to identify residues in the transcription factor of choice, that are necessary to bind a specific DNA motif (Vidal et al., 1996), using the transcription factor fused to the GAL4 activation domain (GAD). The reverse yeast-1-hybrid assay configuration presented here takes advantage of the intrinsic activation activity of a transcription factor to map residues responsible for this transcriptional activation. In this configuration, the transcription factor is fused to the GAL4 DNA-BD (GBD) and the reduction of intrinsic transcriptional activity is achieved by mutating residues potentially involved in interaction with the transcriptional machinery, thus detecting protein–protein interactions instead of DNA–protein interactions (Figure 1A). GAL4-BD fusion constructs have already been used extensively to analyze TADs (Cress and Triezenberg, 1991; Sainz et al., 1997; Yanagisawa, 2001; Titz et al., 2006), though those reports, done in yeast, employed visual screening of selection markers to detect loss of function: LacZ activity for blue/white screening (Estruch et al., 1994; Sainz et al., 1997; Yanagisawa, 2001). This approach is rather laborious, as it requires quantitative assays of individual colonies to determine the loss of function phenotype. The advent of negative selection markers has enabled high throughput screening of proteins with transcriptional activation. One specific use has enabled elimination of auto-activating bait proteins from yeast-2-hybrid libraries, by effectively removing proteins with TADs from high throughput screens (Walhout and Vidal, 1999). By adapting this configuration for use with a single transcription factor instead of a library, we were able to simultaneously screen 1.5 million colonies and map residues responsible for transcriptional activation in PIF3. To our knowledge, this is the first report employing this specific type of screening strategy for systematic analysis of TADs.

One disadvantage of reverse-1-hybrid as well as reverse-2-hybrid screening is that random mutagenesis of the molecule of interest leads to a considerable number of false positive colonies, caused by premature stop codons, overall protein misfolding or low expression in the yeast cells. Such false positives require labor- and cost-intensive methods such as Western blotting to remove them from the analysis (Vidal et al., 1996; Kikis et al., 2009). To overcome this problem, we introduced AUR1C to the screen as a C-terminal fusion with PIF3. This second selectable marker was successfully able to eliminate false positives from the screen (Figure 1C), by monitoring for expression of the full-length fusion product in the presence of AbA. AUR1C encodes an AbA insensitive variant of Inositol-phophorylceramide-synthase, which is critical for Sphingolipid synthesis and can overcome the growth arrest induced by AbA (Hashida-Okado et al., 1996). Since yeast selection usually relies on nutritional auxotrophy, which is limited by the genetic makeup of the yeast strain, AUR1C appears to be a superior selection marker, as it will work in virtually every screening strain, regardless of auxotrophic selection availability. Indeed, none of the colonies sequenced in our screen showed frameshift mutations or premature stop codons (Figure 2A), two of the most common types of false positives, able to persist through consecutive selection steps. It is noteworthy, that we did see plasmids with such defects in colonies bearing more than one plasmid, but these colonies were easily removed from the screen with additional selection steps and did not persist. Additionally, all selected colonies showed reduced transcriptional activity, when compared to the WT-PIF3 (Figure 1C). This underscores the power of AUR1C as a second selectable marker, for eliminating false positives in our reverse-1-hybrid screening strategy.

Yeast based screening methods have the advantage of using homologous recombination to finally assemble the protein of interest into the screening vector, thereby enabling high throughput and simplifying sample preparation (Vo et al., 1997). To facilitate this, the pBLAU plasmid contains a unique SmaI site between the BD and AUR1-C, which linearizes the vector and creates blunt ends, necessary for efficient homologous recombination in yeast (Figure 1D). However, this method still carries a high risk of false positives, since incompletely cut or religated vector without insert expresses BD-AUR1C and would therefore show positive selection on 5FOA and AbA containing plates. To eliminate any potential background from the empty vector in the reverse 1-hybrid assay, it was necessary to clone the BD and AUR1-C out of frame, with the provision that the reading frame of the full length fusion protein could be restored when the protein of interest, and an additional base at the C-terminus, is correctly inserted between the BD and AUR1-C fusion partners (Table 1). This strategy successfully removed the empty vector from our analysis, as all colonies contained the PIF3 insert, as verified by colony PCR. In PIF3, two discrete regions with high mutation co-incidence were detected as well as a few isolated individual residues such as E5, to have an influence on transcriptional activation. Using *in silico* prediction tools, (http://www.med.muni.cz/9aaTAD/), only one TAD, overlapping with the APB motif was predicted to function in transcriptional activation in PIF3. This illustrates the power of the reverse yeast-1-hybrid method presented here to identify novel, unpredicted TADs.

### Functional conservation of TAD

Polymerase II dependent transcription is highly conserved among eukaryotes, both structurally and functionally (Sainsbury et al., 2015). Several transcriptional activators from plants and animals also have transcriptional activity in yeast, demonstrating this functional conservation of the transcriptional activation process between eukaryotes (Sainz et al., 1997; Escher et al., 2000; Yanagisawa, 2001; Li et al., 2013). For example, the herpes virus protein VP16 activates transcription in both, yeast and mammals, although human Med25, a primary interaction partner of VP16 is lacking in yeast cells (Milbradt et al., 2011; Vojnic et al., 2011). Similarly, activation domains specifically from plants have been found to be functional in yeast (Estruch et al., 1994; Sainz et al., 1997; Yanagisawa, 2001; Li et al., 2013). Interestingly, the maize transcription factor C1 displays a quantitatively comparable transcriptional activity between the two organisms, suggesting that the activity in yeast may provide a reasonable indicator of the activity in plants (Sainz et al., 1997). We suggest therefore, that although the functionality of the PIF3 TADs identified here remains to be demonstrated in plants, the data provides initial insight into residues and domains that may be targeted for mutagenesis and testing *in planta*. In addition, the strong suppression of PIF3 activation activity by the double mutant combination of E31V and W96A in yeast, will provide a very useful tool for future screens and assays involving PIF3 interactors.

### Amino acid composition of activation domains in PIF3

Our screen revealed at least two distinct regions with transcriptional activation function in PIF3, which display a comparable potency (Figure 3). Although the significance of multiple TADs in a single activator is not well-understood, this phenomenon has already been observed in several transcription factors, including VP16, p53, and Gcn4 (Ma and Ptashne, 1987; Blair et al., 1994; Jenkins et al., 2009; Herbig et al., 2010). TADs are often autonomously functional and motifs as short as nine residues reportedly show transcriptional activity, when fused to a DNA binding domain (Estruch et al., 1994; Piskacek et al., 2007), this was not the case for PIF3. Neither of the PIF3 TADs alone show significant activity: both need a larger protein context to display any transcriptional activation (Figure 3). Thus, both TADs are necessary, but not sufficient, and their function appears additive rather than synergistic, indicating that the TAD is modular in nature and each module can potentially be regulated independently (Jenkins et al., 2009).

Although most amino acids in TADs are exchangeable without any compromise in function, the hydrophobic amino acids are of crucial importance, and often cannot be replaced even with conserved amino acids (Drysdale et al., 1995), indicating that a minimal sequence specificity for TADs does indeed exist (Cress and Triezenberg, 1991; Warfield et al., 2014). Hydrophobic amino acids, specifically W96 and F110, were also identified in our screen as playing a key role in transcriptional activation (Figure 2C). It is notable that the entire PIF3 sequence contains only two tryptophan residues, both of which are located in the two TADs, and one of which showed the highest mutation occurrence in the entire screen. When mutated to alanine, both W96 and F110 showed a dramatic decrease of transcriptional activity, while still being able to bind phyB in light-dependent fashion (Figure 2C). Therefore, these residues are specifically critical for transcriptional activation. This finding supports the current model of TAD function, in which few hydrophobic amino acids form a minimalistic fuzzy interface between a TAD and the transcriptional machinery (Brzovic et al., 2011; Warfield et al., 2014). Additionally, these hydrophobic residues are conserved in PIF1, PIF4, and PIF5 (Figure 5), and when exchanged for an alanine, transcriptional activation is reduced in these PIFs as well (Figure 6 and Supplementary Figure 1). Surprisingly, PIF5 appears not to be as sensitive to exchanges of this conserved W91 residue or other hydrophobic residues in this domain compared to the other PIFs. It is able to maintain 50% or more transcriptional activity for all tested alanine substitutions in this domain, while displaying strong influence of E31 on transcriptional activity (Figure 6).

Though acidic amino acid residues are not critical for function, they are often prevalent in domains with transcriptional activity (Titz et al., 2006). Noteworthy, the amino acids identified in the screen were also prevalently acidic (E and D) residues, although they do not occur more often in the PIF3 primary sequence: 25% of all 174 identified mutations affected D or E residues and more than two-thirds of all D and E residues in PIF3 were mutated at least once in the screen. Additionally, both identified TADs have multiple conserved acidic residues (Figure 5), classifying them as acidic type TADs. Among the acidic residues affecting transcriptional activation in PIF3, residue E31 is of special interest, because it overlaps with phyB binding (Figure 2C).

### PIF3 activation activity and phyB binding are physically overlapping

The residue E31 in PIF3 shows dual functionality: Exchange to alanine or valine leads to a comparable reduction in transcriptional activation activity, while phyB binding is completely abolished only by the alanine exchange (Figures 2C, 4). On the other hand, exchange into a valine retains phyB interaction. The extent of phyB binding can therefore be quantitatively modulated by the chemical properties of the individual amino acids, and is not an all or nothing response. This modulatability at equivalents to residue E31 is lost in other PIF variants: Both alanine and valine substitutions show no phyB binding (Figure 6 and unpublished data). This could be due to the different binding affinities of the PIFs for phyB. As WT PIF4 and PIF5 bind phyB less efficiently than PIF3 (Khanna et al., 2004), even a small further reduction could suppress detectability in growth assays. This dual activity of E31 is of particular interest, since it raises the question, of whether phyB binding interferes with PIF activation capacity, or whether both can occur simultaneously. We have shown, that yeast grown on chromophore-containing media and co-expressing a wt-PIF3 protein (i.e., no additional fused activation domain), together with a Gal4BD-phyB-N-terminal-domain fusion, shows strong, R-light induced reporter-gene expression, while yeast expressing the phyB N-terminal-domain alone does not (data not shown). Thus, phyB binding and PIF-driven transcriptional activation can occur simultaneously, despite requiring the same residue for both functions in PIF3. Similarly, there is a partial overlap between R-light induced phosphorylation sites and transcriptional activity (Ni et al., 2013), demonstrating another physical overlap of multiple functions on individual residues, challenging the model of discreet functional domains in the protein structure of PIF3.

## Author contributions

PQ: designed research, analyzed data, edited manuscript, edited figures; JD: designed experiments, conducted research, analyzed data, wrote manuscript, assembled figures; UB: conducted experiments, analyzed data, assembled figures; JL: conducted experiments; GC: conducted experiments.

### Conflict of interest statement

The authors declare that the research was conducted in the absence of any commercial or financial relationships that could be construed as a potential conflict of interest.

## Supplementary material

Supplementary Figure 1 **PIF3 transcriptional activity and expression levels. (A)** Absolute Miller units measured for three biological replicas of LexA-PIF3. Each biological replica was measured with two technical repeats. **(B)** Shown are the expression levels of PIF3 variants as measured in a Western blot against LexA. **(C)** Relative Miller unit activity of PIF3 and its variants in %. Data is the mean value of data shown in panel **(A)**, error bars indicate standard error of the mean.

Supplementary Figure 2 **PIF1 transcriptional activity and expression levels. (A)** Absolute Miller units measured for three biological replicas of LexA-PIF1. Each biological replica was measured with three technical repeats. **(B)** Shown are the expression levels of PIF1 variants as measured in a Western blot against LexA. **(C)** Relative Miller unit activity of PIF1 and its variants in %. Data is the mean value of data shown in panel **(A)**, error bars indicate standard error of the mean. This panel is a complete depiction of the relative Miller unit activity shown in Figure 6B.

Supplementary Figure 3 **PIF4 transcriptional activity and expression levels. (A)** Absolute Miller units measured for four biological replicas of BD-PIF4-AUR1C. Each biological replica was measured with three technical repeats. **(B)** Shown are the expression levels of PIF4 variants as measured in a Western blot against BD.

Supplementary Figure 4 **PIF5 transcriptional activity and expression levels. (A)** Absolute Miller units measured for four biological replicas of BD-PIF5-AUR1C. Each biological replica was measured with three technical repeats. **(B)** Shown are the expression levels of PIF5 variants as measured in a Western blot against BD.

Supplementary Table 1 **Primers and their sequences used for cloning**.
