# Supplementary figures and images for: A Modified Reverse One-Hybrid Screen Identifies Transcriptional Activation Domains in PHYTOCHROME-INTERACTING FACTOR 3
Source: Front Plant Sci. 2016 Jun 17;7:881. doi: 10.3389/fpls.2016.00881 (PMC4911399; doi:10.3389/fpls.2016.00881)

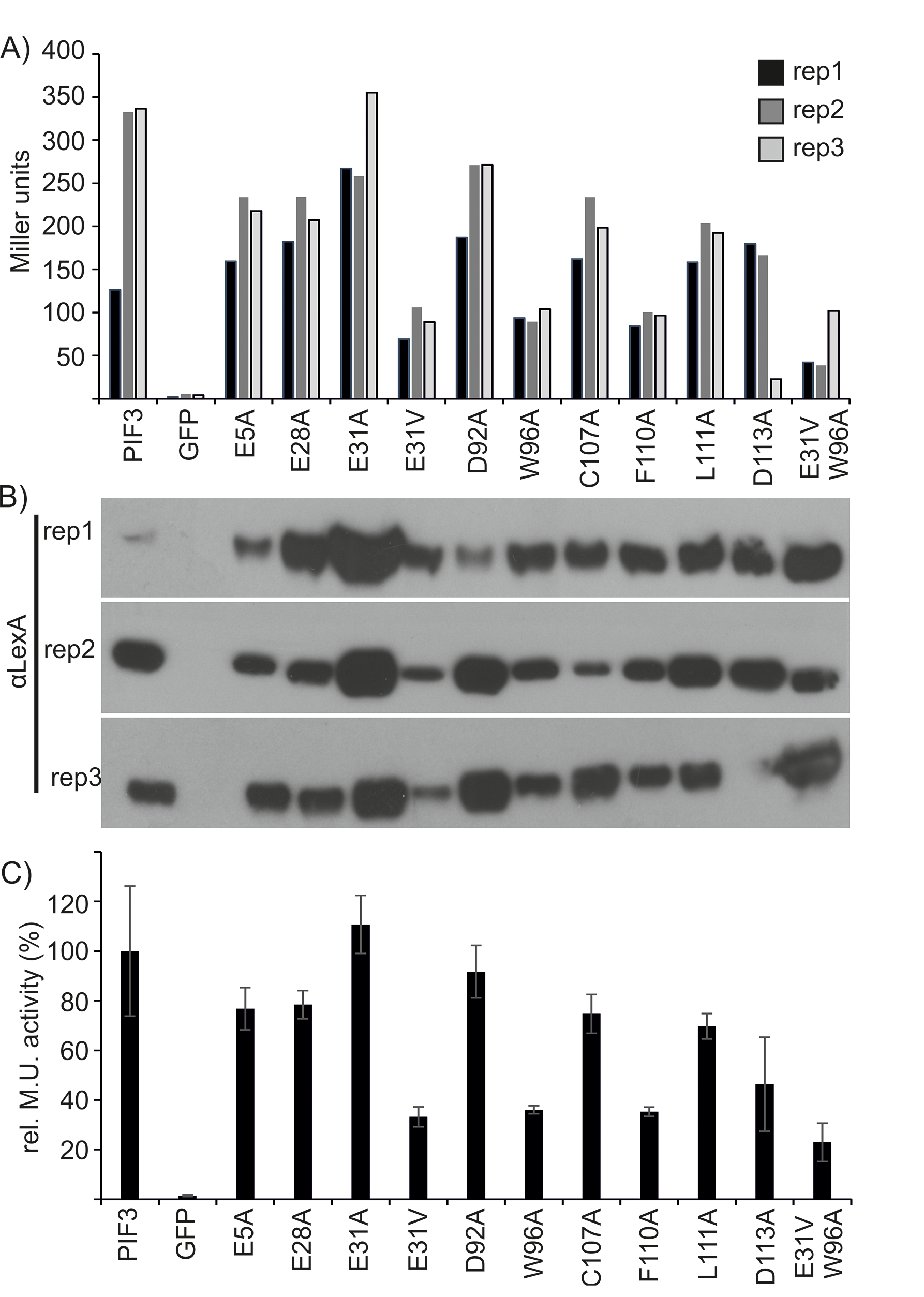

Supplement: Supplementary Figure 1 — PIF3 transcriptional activity and expression levels. (A) Absolute Miller units measured for three biological replicas of LexA-PIF3. Each biological replica was measured with two technical repeats. (B) Shown are the expression levels of PIF3 variants as measured in a Western blot against LexA. (C) Relative Miller unit activity of PIF3 and its variants in %. Data is the mean value of data shown in panel (A), error bars indicate standard error of the mean. [file Image1.TIF]

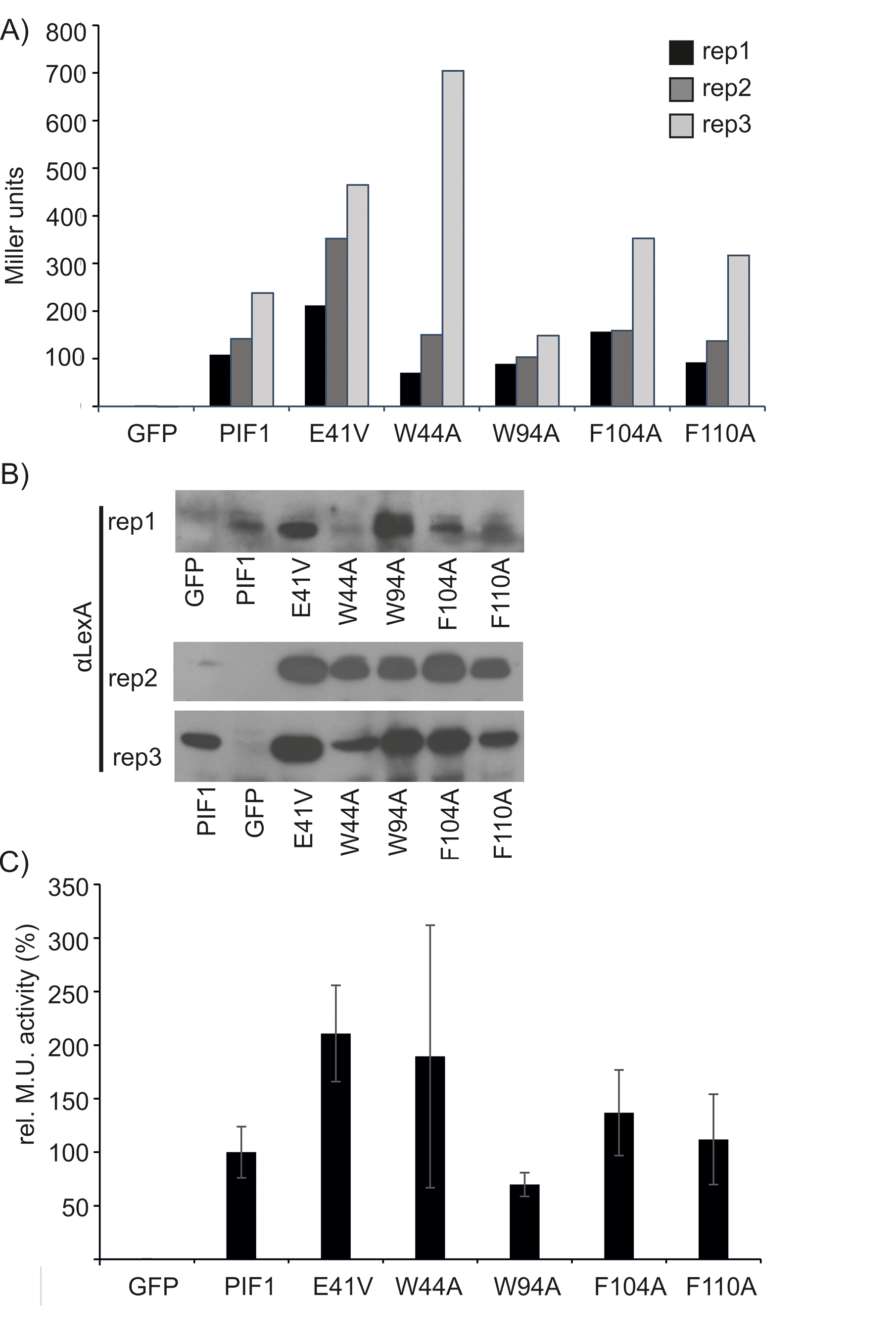

Supplement: Supplementary Figure 2 — PIF1 transcriptional activity and expression levels. (A) Absolute Miller units measured for three biological replicas of LexA-PIF1. Each biological replica was measured with three technical repeats. (B) Shown are the expression levels of PIF1 variants as measured in a Western blot against LexA. (C) Relative Miller unit activity of PIF1 and its variants in %. Data is the mean value of data shown in panel (A), error bars indicate standard error of the mean. This panel is a complete depiction of the relative Miller unit activity shown in Figure 6B. [file Image2.TIF]

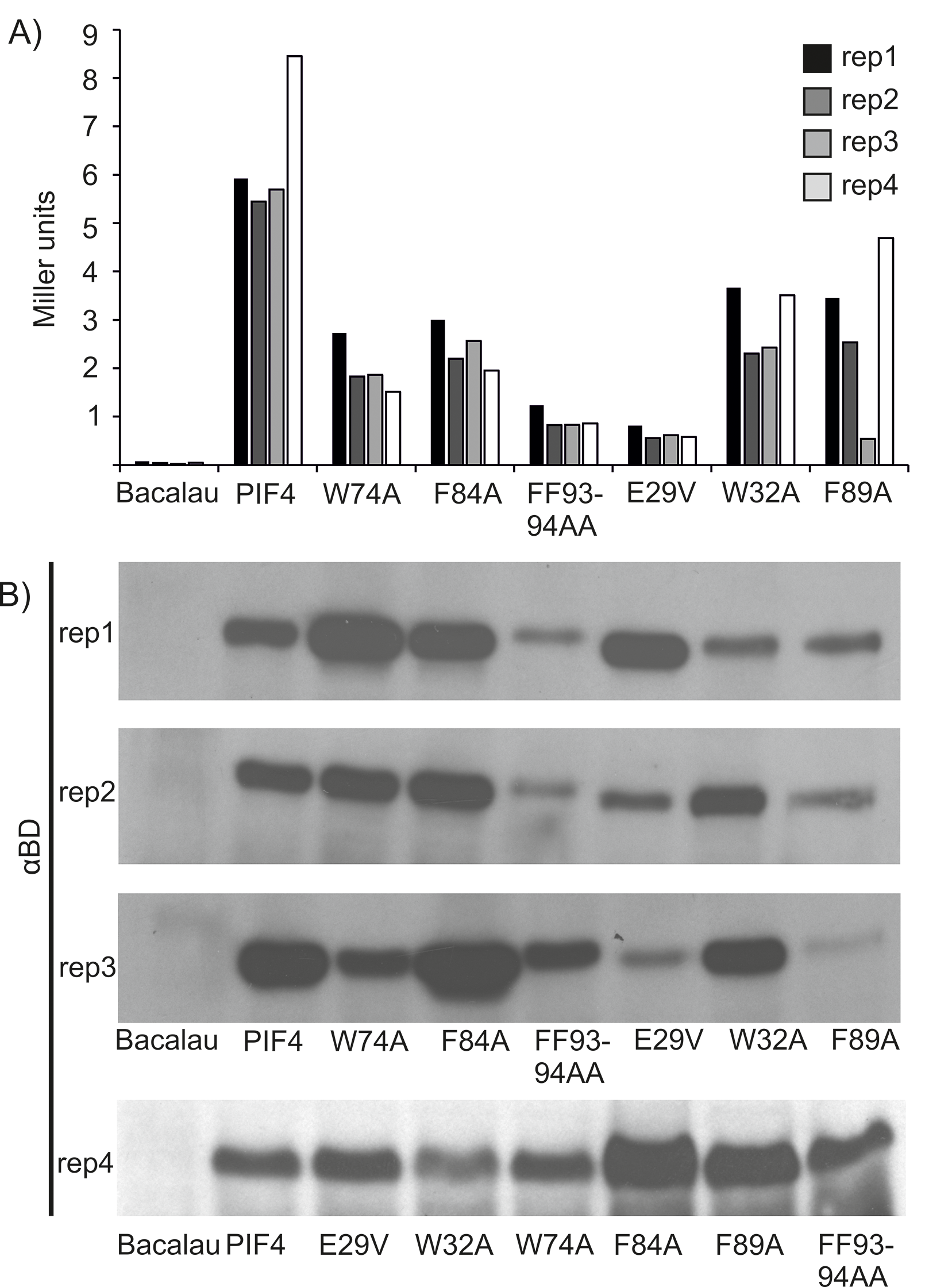

Supplement: Supplementary Figure 3 — PIF4 transcriptional activity and expression levels. (A) Absolute Miller units measured for four biological replicas of BD-PIF4-AUR1C. Each biological replica was measured with three technical repeats. (B) Shown are the expression levels of PIF4 variants as measured in a Western blot against BD. [file Image3.TIF]

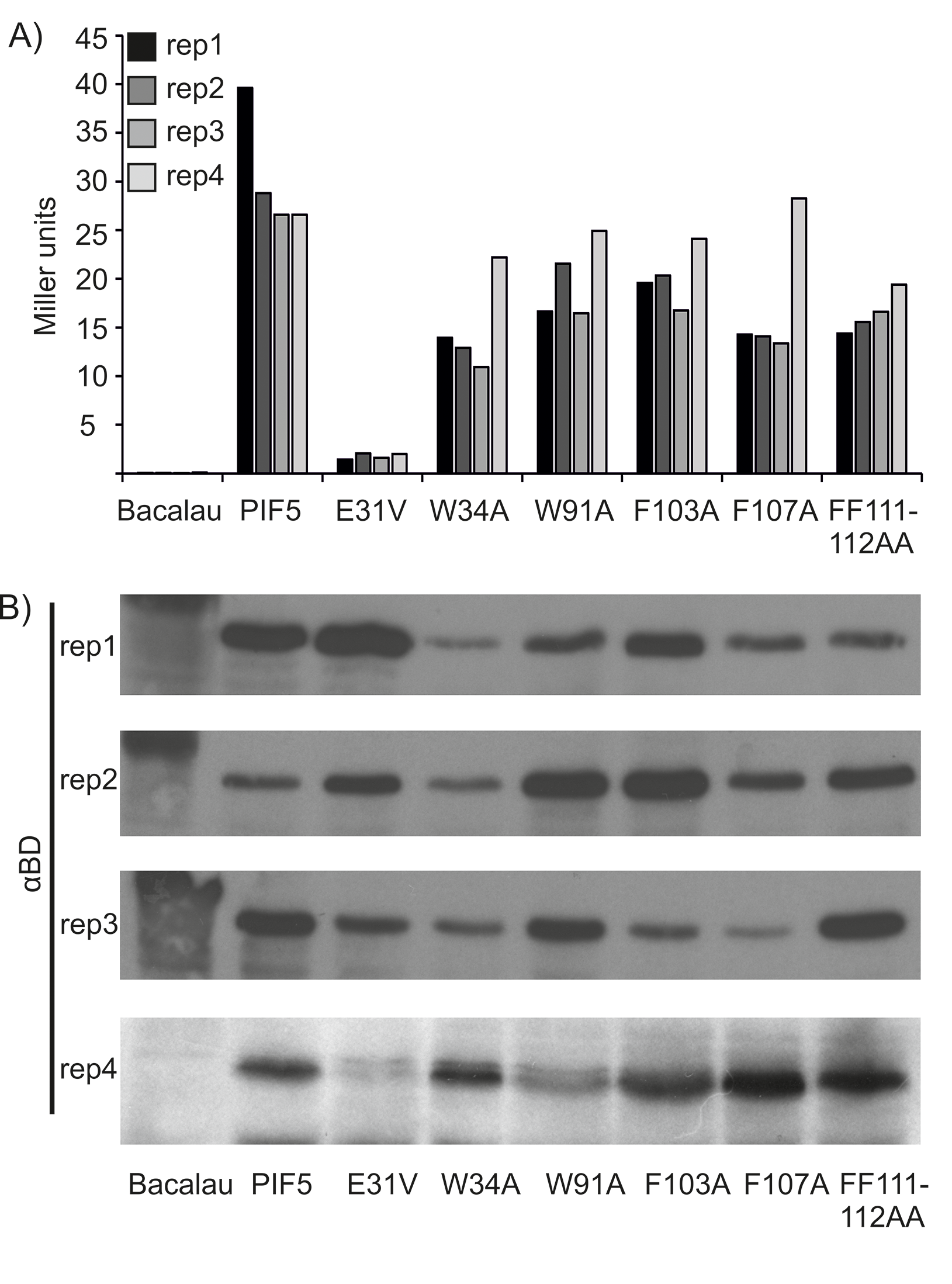

Supplement: Supplementary Figure 4 — PIF5 transcriptional activity and expression levels. (A) Absolute Miller units measured for four biological replicas of BD-PIF5-AUR1C. Each biological replica was measured with three technical repeats. (B) Shown are the expression levels of PIF5 variants as measured in a Western blot against BD. [file Image4.TIF]
