# Supplementary material for: A Modified Reverse One-Hybrid Screen Identifies Transcriptional Activation Domains in PHYTOCHROME-INTERACTING FACTOR 3
Source: Front Plant Sci. 2016 Jun 17;7:881. doi: 10.3389/fpls.2016.00881 (PMC4911399; doi:10.3389/fpls.2016.00881)
Supplement: Supplementary Table 1 — Primers and their sequences used for cloning. [file Table1.DOCX]

**Supplemental table 1: Primers and their sequences used for cloning**

| MZ380 |  | CTCCAAGCTTGAAGCAAGCCTCCTGAGCTCATACAATTAATTAAGATGAAGCTACTGTCTTCTATCGAACAA |
| --- | --- | --- |
| MZ381 |  | TTGATACTAGTTCACGATACAGTCAACTGTCTTTGACCTTTG |
| MZ382 |  | TTGATACTAGTCCGTCGACCTGCAGCCAAGCTAATTC |
| MZ383 |  | GAGCCCCGAAAGCTTACATTTTATG |
| JR137 |  | AAGAGCTCGGAGTTGATTGTATGCTTGGT |
| JR138 |  | CCGAGCTCATCCTTTTGTTGTTTCCGGGT |
| JR189 |  | TTAATTAATGGCCCGGGAAGCAAGCTATGAAGCTACTGTCTTCTATCGA |
| JR190 |  | ACTAGTACACGTATACTCACGTGCACAATAGTCACCCGATACAGTCAACTGTCTTTGA |
| JR191 |  | ACTAGTATGGCAAACCCTTTTTCGAGATGGT |
| JR192 |  | GTCGACTTAAGCCCTCTTTACACCTAGTGAC |
| JR195 |  | AATTAATTAATGGAAGCAAGCTATGAAGCTACTGTCTTCTATCGAA |
| JR196 |  | ATACTAGTCCACACGTATACCCACGTGCCCCGGGACAATAGTCACCCGATACAGTCAACTGTCTTTGACC |
| JR197 |  | GCCATACTAGTCCACACGTATACCCACGTGCCCCACGACGATCCACAAAACTGATCAGAA |
| JR198 |  | GTCAAAGACAGTTGACTGTATCGGGTGACTATTGTCCCATGCCTCTGTTTGAGCTTTTCAGGC |
| JR200 |  | ATCCCGGGATGCCTCTGTTTGAGCTTTTCA |
| JR205 |  | CCCGGGACGACGATCCACAAAACTGAT |
| JR226 |  | CCCGGGATGCATCATTTTGTCCCTGAC |
| JR227 |  | CCCGGGTACCTGTTGTGTGGTTTCCGTG |
| JR228 |  | CCCGGGATGGAACACCAAGGTTGGAGT |
| JR229 |  | CCCGGGTGTGGTCCAAACGAGAACCGTC |
| JR230 |  | CCCGGGATGGAACAAGTGTTTGCTGAT |
| JR231 |  | CCCGGGTGCCTATTTTACCCATATGAAG |
| JL01 | PIF1 W94A fw | CAAGAAGATGAAATGACTTCTGCGCTTCATTATCCTCTCCGTGACG |
| JL02 | PIF1 W94A rv | CGTCACGGAGAGGATAATGAAGCGCAGAAGTCATTTCATCTTCTTG |
| JL03 | PIF1 F104A fw | CCTCTCCGTGACGATGATGCCTGCTCAGATCTTCTC |
| JL04 | PIF1 F104A rv | GAGAAGATCTGAGCAGGCATCATCGTCACGGAGAGG |
| JL05 | PIF1 F110A fw | CTGCTCAGATCTTCTCGCCTCCGCCGCACCTACTGCG |
| JL06 | PIF1 F110A rv | CGCAGTAGGTGCGGCGGAGGCGAGAAGATCTGAGCAG |
| JR246 | PIF1 W44Afw | GATGATCTTATGGAGCTTTTAGCGCAGAACGGTCAAGTTGTTGTT |
| JR247 | PIF1 W44A rv | AACAACAACTTGACCGTTCTGCGCTAAAAGCTCCATAAGATCATC |
| JL25 | PIF1 E41V Fwd | GTGAAGATGATGATCTTATGGTGCTTTTATGGCAGAACGGTCAAGTTG |
| JL26 | PIF1 E41V Rev | CAACTTGACCGTTCTGCCATAAAAGCACCATAAGATCATCATCTTCAC |
| JL07 | PIF 4 W74A fw | GAAGATCAAGAAACTGTCTCTGCGATCCAATACCCTCCAGATGAAG |
| JL08 | PIF4 W74A rv | CTTCATCTGGAGGGTATTGGATCGCAGAGACAGTTTCTTGATCTTC |
| JL09 | PIF4 F84A fw | CCTCCAGATGAAGACCCAGCCGAACCCGACGACTTC |
| JL10 | PIF4 F84A rv | GAAGTCGTCGGGTTCGGCTGGGTCTTCATCTGGAGG |
| JL27 | PIF4 E29V Fwd | CAGGCCACAAGATGAACTAGTGGTGTTATTATGGCGAGATGGACAAG |
| JL28 | PIF4 E29V Rev | CTTGTCCATCTCGCCATAATAACACCACTAGTTCATCTTGTGGCCTG |
| JR252 | PIF4 F89A fw | CCATTCGAACCCGACGACGCCTCCTCCCACTTCTTCTCA |
| JR253 | PIF4 F89A fw | TGAGAAGAAGTGGGAGGAGGCGTCGTCGGGTTCGAATGG |
| JR254 | PIF4 FF93-94AA fw | GAACCCGACGACTTCTCCTCCCACGCCGCCTCAACCATGGATCCCCTCCAGAGA |
| JR255 | PIF4 FF93-94AA rv | TCTCTGGAGGGGATCCATGGTTGAGGCGGCGTGGGAGGAGAAGTCGTCGGGTTC |
| JR244 | PIF4 W32A fw | GAACTAGTGGAGTTATTAGCGCGAGATGGACAAGTGGTTCTG |
| JR245 | PIF4 W32A rv | CAGAACCACTTGTCCATCTCGCGCTAATAACTCCACTAGTTC |
| JL15 | PIF5 W91A fw | GATGATCAAGAAACCGTCTCCGCGATACAATACCCTCCGGATGACG |
| JL16 | PIF5 W91A rv | CGTCATCCGGAGGGTATTGTATCGCGGAGACGGTTTCTTGATCATC |
| JL17 | PIF5 F103A fw | GGATGACGTCATCGACCCTGCCGAATCCGAGTTCTCCTCTC |
| JL18 | PIF5 F103A rv | GAGAGGAGAACTCGGATTCGGCAGGGTCGATGACGTCATCC |
| JL19 | PIF5 FF111-112AA fw | CCGAGTTCTCCTCTCATGCCGCCTCTTCGATCGATCACCTCGGAGG |
| JL20 | PIF5 FF111-112AA rv | CCTCCGAGGTGATCGATCGAAGAGGCGGCATGAGAGGAGAACTCGG |
| JL21 | PIF5 F107A fw | CCTGCCGAATCCGAGGCCTCCTCTCATGCCGCCTC |
| JL22 | PIF5 F107A rv | GAGGCGGCATGAGAGGAGGCCTCGGATTCGGCAGG |
| JL29 | PIF5 E31V fw | CAGACCAGAAGATGAATTAGTGGTGCTATTGTGGAGAGATGGTCAAG |
| JL30 | PIF5 E31V fw | CTTGACCATCTCTCCACAATAGCACCACTAATTCATCTTCTGGTCTG |
| JR248 | PIF5 W34A fw | GATGAATTAGTGGAGCTATTGGCGAGAGATGGTCAAGTGGTTTTA |
| JR249 | PIF5 W34A rv | TAAAACCACTTGACCATCTCTCGCCAATAGCTCCACTAATTCATC |
| UB03 | PIF3 E5A fw | GGATGCCTCTGTTTGCGCTTTTCAGGCTCAC |
| UB04 | PIF3 E5A rv | GTGAGCCTGAAAAGCGCAAACAGAGGCATCC |
| UB07 | PIF3 E28A fw | CTCCACCTGTAGATGCAGTTGTGGAGCTGG |
| UB08 | PIF3 E28A rv | CCAGCTCCACAACTGCATCTACAGGTGGAG |
| UB11 | PIF3 D92A fw | GGTTTGAGTCAAGACGATGCCTTTGTTCCATGGTTGAATC |
| UB12 | PIF3 D92A rv | GATTCAACCATGGAACAAAGGCATCGTCTTGACTCAAACC |
| UB15 | PIF3 W96A fw | GACGATGACTTTGTTCCAGCGTTGAATCATCATCCCTCC |
| UB16 | PIF3 W96A rv | GGAGGGATGATGATTCAACGCTGGAACAAAGTCATCGTC |
| UB19 | PIF3 C107A fw | CCCTCCCTTGATGGATATGCCTCTGATTTCTTGCGTG |
| UB20 | PIF3 C107A rv | CACGCAAGAAATCAGAGGCATATCCATCAAGGGAGGG |
| UB25 | PIF3 E31A fw | GTAGATGAAGTTGTGGCGCTGGTGTGGGAAAATG |
| UB26 | PIF3 E31A rv | CATTTTCCCACACCAGCGCCACAACTTCATCTAC |
| UB31 | PIF3 F110A fw | GATGGATATTGCTCTGATGCCTTGCGTGATGTGTCGTC |
| UB32 | PIF3 F110A rv | GACGACACATCACGCAAGGCATCAGAGCAATATCCATC |
| UB33 | PIF3 L111A fw | GATATTGCTCTGATTTCGCGCGTGATGTGTCGTCTCC |
| UB34 | PIF3 L111A rv | GGAGACGACACATCACGCGCGAAATCAGAGCAATATC |
| UB37 | PIF3 D113A fw | CTCTGATTTCTTGCGTGCTGTGTCGTCTCCTGTTAC |
| UB38 | PIF3 D113A rv | GTAACAGGAGACGACACAGCACGCAAGAAATCAGAG |
| JR388 | PIF3 E31V fw | CCTGTAGATGAAGTTGTGGTGCTGGTGTGGGAAAATGGT |
| JR389 | PIF3 E31V rv | ACCATTTTCCCACACCAGCACCACAACTTCATCTACAGG |
